# Freedom of movement and health of nursing home residents with dementia: an exploratory cross-sectional study

**DOI:** 10.1186/s12877-025-05774-3

**Published:** 2025-02-22

**Authors:** Suzan P. van Liempd, Sascha R. Bolt, Katrien G. Luijkx

**Affiliations:** 1 Department of Tranzo, Scientific Centre for Care and Wellbeing, School of Social and Behavioral Sciences, Tilburg University, Tilburg, The Netherlands; 2 Stichting Mijzo, Waalwijk, the Netherlands

**Keywords:** Freedom of movement, Open doors, Nursing homes, Dementia, Health

## Abstract

**Background:**

Having more freedom of movement may relate to better health in nursing home (NH) residents with dementia. Research that tests whether residents in NHs with more freedom of movement are healthier compared to residents in closed NHs is scarce. Also, existing research on freedom of movement does not consider the diverse dimensions of health. This study explored health differences between two groups of nursing home residents with dementia with different levels of freedom of movement.

**Methods:**

We used a quantitative cross-sectional design to investigate differences in health between two groups of NH residents with dementia. One group lived in closed NHs (i.e., with closed unit doors) and the other group in semi-open NHs (i.e., with closed NH entrance doors). A total of 124 residents with dementia were recruited from five NHs in the Netherlands, of whom 61 residents lived in semi-open NHs and 63 residents lived in closed NHs. Data were collected using questionnaires to cover health dimensions according to the concept of Positive Health, including quality of life and participation, mental functioning and perception, daily functioning and bodily functions. An analysis of covariance, adjusted for age, gender and type of dementia, was used to examine differences in residents’ health.

**Results:**

Most included residents had Alzheimer’s or vascular dementia and 68% were female. No significant demographic differences were observed between the two groups in age, gender, type of dementia, length of stay, length of diagnoses and type of care package (*p*-values ranged from 0.097 to 0.606). After adjusting for multiple comparisons, there were no significant differences in any of the assessed health dimensions between residents of semi-open nursing homes and those of closed nursing homes, with a significance threshold of *p* <.004 accounting for the correction for multiple testing (*p*-values ranged from 0.020 to 0.870).

**Conclusions:**

This exploratory study found no significant differences in health between residents with dementia in semi-open and closed NHs. These findings contradict earlier research suggesting that more freedom of movement may enhance overall health in this population. Further research, preferably employing longitudinal designs, is necessary to establish causal pathways and identify the underlying mechanisms.

## Background

The design of the physical environment of a nursing home (NH) is increasingly recognized as important in the care for persons with dementia and for the management of their behaviors [1–3]. For NH residents with dementia, the physical environment is often restricted by means of a closed-door policy of the NH, which limits their freedom of movement [4, 5]. Freedom of movement involves the right to (decide to) independently move from one place to another [6]. NHs enforce different levels of freedom of movement. In a closed NH, residents with dementia are free to move within a unit but are not allowed to independently leave this unit without supervision. In a semi-open NH, residents can move freely within the facility, including enclosed areas such as gardens, but are not allowed to enter the outside world independently. In an open NH, residents can go wherever they want, not hindered by closed entrance doors [6].

A closed living environment can make NH residents with dementia or their caregivers feel safe and secure [7]. However, living behind closed doors also conflicts with the right to freedom of movement [8]. Moreover, it may be associated with negative health outcomes for residents, such as higher levels of agitation and social isolation [8, 9]. A prior scoping review indicates that restricted freedom of movement by locked doors is commonly reported as a significant source of frustration for NH residents with dementia [10]. As a result, residents may exhibit resistance or distress; behaviors that are frequently characterized as challenging or as behavioral and psychological symptoms of dementia [9, 10].

A previous systematic review suggested that when freedom of movement is increased, for example, by adding a freely accessible enclosed garden, the health of NH residents with dementia may improve [6]. Additionally, in their meta-synthesis, Førsund and colleagues found that the feeling of having freedom of movement is very important for people living with dementia [11], and that being able to open doors may give them a sense of autonomy [12]. This positive relationship between increased freedom of movement and health was also observed in a recent study that followed residents who relocated from a closed nursing home to a semi-open nursing home. Among other outcomes, the quality of life and levels of agitation significantly improved in the semi-open NH compared to the closed NH [13]. Despite the positive relationship between freedom of movement and the health of NH residents with dementia, the balance often tips toward safety, possibly resulting in the continued locking of entrance doors [4, 5].

Due to the possible negative relationship between health and living behind closed doors, there may be differences between the health of residents living in closed NHs and residents living in NHs that offer more freedom of movement. However, scientific research that tests the assumption that residents in NHs with more freedom of movement are healthier compared with residents in closed NHs is scarce. Moreover, existing studies that examined health outcomes of NH residents with different levels of freedom of movement, have concentrated mostly on single aspects of health, such as on residents’ physical health and quality of life [9]. Although these health aspects are important parameters in the assessment of relationships between health and freedom of movement, residents with dementia with different levels of freedom of movement may differ on several other important health dimensions. According to the concept of positive health, which entails a holistic approach, health also includes social and societal participation, mental functions and perception, daily functioning and existential health [14]. Hence, research is needed to investigate differences between residents in NHs that offer different levels of freedom of movement, with respect to the various health dimensions. Therefore, the current exploratory study investigates whether health differs between NH residents with dementia living in closed NHs and those living in NHs with more freedom of movement.

## Methods

### Study design and ethics

We used a quantitative cross-sectional design to investigate differences in health among two groups of NH residents with dementia. One group lived in closed NHs, and the other group lived in semi-open NHs. This study was approved by the Ethics Review Board of Tilburg University (reference Amendment RP241) and the Research and Science Committee of the participating care organization.

### Participants and setting

Residents of five NHs were recruited from 5 May to 31 May 2021, of which two NHs are semi-open (NH A and B) and three NHs are closed (NH C, D and E). The NHs were locations of the same non-profit, publicly funded long-term care organization in the Netherlands. The involved NHs fit the international definition, namely: a facility with a domestic-styled environment that provides 24-hour functional support and care for persons who require assistance with activities of daily living (ADL) and who often have complex health needs and increased vulnerability [15]. These five locations were selected because they accommodate the same target group, namely individuals with dementia. The locations of this long-term care organization adhere to a consistent care concept, encompassing uniform staffing levels and a shared vision on care provision. Together, these locations provided a sufficiently large study population, with residents evenly distributed between closed and semi-open NHs.

All 161 residents living in these five NHs had a diagnosis of dementia and were therefore eligible for inclusion. Legal representatives of these residents received written information about the study and were asked to provide written consent if they agreed with the resident’s participation in this study. Responses were stored in a secure, encrypted database accessible only to the lead researcher. All data were processed completely anonymously and treated confidentially in accordance with ethical guidelines and data protection regulations. To protect the privacy of residents, personal data were dissociated from the collected information. In this way, the data were not re-identifiable and could not be traced back to residents in any way.

NH A is a small-scale apartment complex for 26 residents with dementia with 587 m² of common space and an enclosed garden with terrace surrounded by plants and greenery (22.6 m²/resident). There are four residential groups of which two groups are situated on the first floor and two groups are situated on the second floor. A residential group consists of six to eight residents. Each residential group has its own living room where residents spend most of their time together. In this NH, residents have freedom of movement within the building and adjacent garden. All interior doors and the door that gives access to the enclosed garden are open for the largest part of the day. Residents are free to use the elevator that connects the two floors.

NH B is a large NH and has room for 60 residents with dementia, spread across three floors with 2346 m² of common space and with various enclosed gardens, plazas, benches and terraces (39.1 m²/resident). The first floor consists of a large plaza with a kiosk and seven living rooms, each with a different theme. In this NH, all residents are free to move within the building and enter the surrounding, enclosed gardens. Residents are free to use the elevator that connects the three floors.

NH C is a large NH and on the first floor, there are two closed units that each accommodate 25 residents with dementia. Each of the units consists of 547 m² of common space and was adjacent to an enclosed garden (21.9 m²/resident). Each unit has a continuous corridor along which single or double rooms and four living rooms are located. Residents cannot go outside the unit by themselves, as the front entrance of the unit has a coded door.

NH D resides in a large building with on the fourth floor 20 apartments for residents with dementia with 227 m² of common space (11.4 m²/resident). These 20 apartments face one corridor, bordered on either side by a closed door. Two living rooms are located in the middle of the corridor, one of which has a small balcony. Residents cannot go freely outside because of the coded doors at both sides of the corridor.

NH E is a large NH of which one group consists of residents with dementia, who reside on a protected unit with 380 m² of common space. The unit for residents with dementia consists of 14 apartments, a walking loop and two mutual living rooms with a small courtyard garden surrounded by walls (27.1 m²/resident). Residents with dementia cannot independently leave the unit, which has a coded door.

In this study, we define NHs A and B as semi-open; residents with dementia can move freely within the facility, including enclosed areas such as gardens, but are not allowed to enter the outside world independently. NHs C, D and E are defined as closed; residents with dementia are free to move within a unit but are not allowed to independently leave this unit without supervision’.

### Measures and procedure

First, resident characteristics were gathered by the researcher from the care record of each resident. These characteristics were age, gender, type of dementia, length of stay in a NH, length of diagnosis and type of care intensity package. A care intensity package is a Dutch proxy for the intensity of NH care that the resident needs which is assessed by the Care Need Assessment Centre. Second, to investigate the residents’ overall health, different questionnaires were applied that covered the dimensions of Positive Health [14]. Positive Health is defined as an approach that emphasizes enhancing an individual’s overall well-being and functioning, rather than focusing solely on the absence of disease. It underscores the capacity of individuals to adapt, self-manage, and thrive despite their health challenges [16]. This concept encompasses six dimensions: bodily functions, mental functioning and perception, quality of life, social and societal participation, daily functioning, and the existential dimension [14]. To measure these dimensions, we utilized available instruments. Quality of life, participation and the existential dimension were measured using the Qualidem [17], which includes nine subscales relevant to individuals with dementia [18]. The reliability of the Qualidem subscales varies. It is good to excellent for positive affect, positive self-image, care relationship, and negative affect; questionable to acceptable for restless tense behavior, social relations, social isolation, and feeling at home; and poor for having something to do [18]. Scores on the Qualidem subscales were calculated as the mean of items on a 4-point Likert scale (0 = never, 3 = almost every day), with negatively worded items reverse-coded. Higher scores indicate better quality of life. Mental functioning and perception were measured using the Cohen-Mansfield Agitation Inventory (CMAI) [19], the Cornell Scale for Depression in Dementia (CSDD) [20] and the InterRAI Long Term Care Facilities System (interRAI LTFC) section C, Cognition [21]. The CMAI is a valid and reliable measure of agitation [22]. The CMAI score represents the mean of 29 agitated behaviors, each rated on a 7-point Likert scale, ranging from 1 (never) to 7 (several times an hour). Higher scores indicate greater agitated behavior. The CSDD is a reliable instrument for assessing depression among nursing home residents with dementia [23] and has shown good validity [24, 25]. The CSDD score is calculated as the sum of 19 items on a 3-point Likert scale (0 = absent, 1 = slight or variable presence, 2 = severe). Scores of 8 or higher suggest mild depression, while scores of 12 or higher indicate moderate to severe depression. The interRAI LTFC provides a comprehensive geriatric assessment of health and functional domains, including cognition, with demonstrated adequate reliability across various long-term care settings [26]. For the InterRai Cognition scale, lower scores reflect better cognitive function. A score of 0 indicates independence with good memory, whereas scores between 1 and 5 indicate (to a lesser or greater extent) coma, memory problems, or worsening cognitive function. Daily functioning was assessed with the Barthel Index [27] which has acceptable internal consistency for evaluating residents with dementia [28]. The Barthel Index is the mean of 20 items scored 0 (dependent/incapable/incontinent) or 1 (independent/capable/continent), with higher scores indicating greater independence in basic ADL and mobility. A score of 20 indicates complete independence, 15–19 represents fairly to considerably independent, 10–14 indicates needing help but managing a lot independently, 5–9 suggests severe dependence on assistance, and 0–4 reflects complete dependence. Bodily functions, in particular balance and mobility, was measured using the Performance-Oriented Mobility Assessment according to Tinetti (POMA) [29] which has acceptable predictive validity for fall risk and good inter-rater reliability [30]. The POMA score is calculated as the mean of 25 items on a 3-point Likert scale, ranging from 0 (instability or deviation) to 3 (stability or normalcy). Lower scores reflect greater mobility problems, with scores below 19 associated with a five-fold increased risk of falls. Lastly, we used the OAZIS-Dementia questionnaire to describe the physical environment of the two settings (closed and semi-open NH) [31, 32]. The OAZIS-Dementia has previously demonstrated strong inter-rater reliability [31]. The OAZIS-Dementia score represents the average per unit-level across eight categories, comprising a total of 78 items rated on a 5-point Likert scale (1 = strongly disagree, 5 = strongly agree). Higher scores indicate a more positive and supportive environment for residents.

Two care professionals per unit, who were closely involved in the care for the included residents, completed in pairs the Qualidem, CMAI, InterRAI LTFC section C and Barthel index for each resident digitally using a laptop. The CSDD was administered to each resident by a psychologist and care professional per unit. The POMA was performed by a physical therapist per unit for each resident who was willing and could physically perform the test. Prior to the study, the researcher provided each involved care professional with written information about the research and a description of their role. Each questionnaire included detailed instructions on how to complete it. The researcher met with each pair of care professionals to provide additional explanations about the questionnaire if needed. All pairs indicated that they understood the questionnaire and had no questions. The OAZIS-dementia was completed for each NH by the care manager, the facilities manager and a care professional. Any discrepancies were discussed and resolved, under the guidance of the researcher. All measurements took place from 1 to 30 June 2021.

### Analysis

Demographic characteristics of included residents were described by calculating means with standard deviations for continuous data or frequencies for categorical data. T-tests and chi squared tests were used to compare the demographic characteristics of residents of semi-open NHs and residents of closed NHs. The physical environments’ categories were analyzed using descriptive statistics to calculate the means and standard deviations. T-tests were used to determine the statistical significance of the differences observed. Unadjusted group differences in health were explored with t-tests. We used analysis of covariance (ANCOVAs) to adjust for age, gender, and type of dementia. To control for the risk of type I errors, we applied the Bonferroni correction for multiple testing, adjusting the significance level to *p* <.004. Data were analyzed using SPSS version 28.0.

## Results

### Resident and environment characteristics

Of the 161 eligible residents, legal representatives of 124 residents (77%) agreed with study participation and provided written informed consent. Resident characteristics are presented in Table 1. Women comprised 67.7% of the overall study population, with 74.6% residing in closed NHs and 60.5% in semi-open NHs (*p* =.097). The age of residents ranged from 63 to 99 years, with a mean age of 83.6 years. In semi-open NHs, the mean age was 82.9 years (SD = 7.5), while in closed NHs, it was 84.3 years (SD = 7.2) (*p* =.296). Residents predominantly had Alzheimer’s disease (29%) or vascular dementia (29%), with similar distributions in both semi-open and closed NHs (*p* =.314). The average length of stay was 22.5 months (SD = 23.8), with residents in semi-open NHs staying for 24.1 months (SD = 26.4) and those in closed NHs for 20.9 months (SD = 21.1) (*p* =.460). The mean duration of diagnoses was 4.32 years (SD = 3.2), with residents in semi-open NHs having a duration of 4.6 years (SD = 3.6) and those in closed NHs 3.9 years (SD = 2.7) (*p* =.221). ZZP 5 care packages were predominant, with 81.5% of all residents receiving this level of care. In semi-open NHs, 83.3% of residents received ZZP 5 care packages, compared to 79.7% in closed NHs (*p* =.606).

**Table 1:** Characteristics of residents in closed and semi-open settings

Characteristics	Total *n*, mean (SD)/*n* (%)	Semi-open NHs *n*, mean (SD)/*n* (%)	Closed NHs *n*, mean (SD)/*n* (%)	*p*-value
Age	124; 83.6 (7.3)	61; 82.9 (7.5)	63; 84.3 (7.2)	0.296
Gender (% female)	84 (67.7%)	37 (60.7%)	47 (74.6%)	0.097
Type of dementia				0.314
Alzheimer	36 (29%)	16 (26.2%)	20 (31.7%)	
Vascular dementia	36 (29%)	18 (29.5%)	18 (28.6%)	
Vascular/mixed dementia	21 (16.9%)	14 (23%)	7 (11.1%)	
Other types of dementia	31 (25%)	13 (21.3%)	18 (28.6%)	
Length of stay (months)	124; 22.5 (23.8)	61; 24.1 (26,4)	63; 20.9 (21.1)	0.460
Length of diagnoses (in years)	115; 4.32 (3.2)	56; 4.6 (3.6)	59; 3.9 (2.7)	0.221
Type of care package				0.606
ZZP 5	97 (81.5%)	50 (83.3%)	47 (79.7%)	
ZZP 7	22 (18.5%)	10 (16.7%)	12 (20.3%)	

N: number of participants; SD: standard deviation; Type of care package; ZZP 5: Sheltered living with intensive dementia care; ZZP 7: sheltered living with very intensive care due to specific conditions, with emphasis on supervision

Table 2 depicts the mean scores for each category of the OAZIS-Dementia by nursing home type, along with the total scores. On average, both types of NHs scored above 3 on the OAZIS-dementia categories. This indicates high overall scores on the NHs’ physical and supportive environmental aspects. Significant differences between the semi-open and closed NHs were found in three categories: Windows and views (*p* =.034), Interior (*p* =.032), and Staff (*p* =.015).

**Table 2:** Scores on the OAZIS-Dementia per type of nursing home

Categories (number of items)	Semi-open NHs (*n* = 2)			Closed NHs (*n* = 3)		
	Mean	SD		Mean	SD	*p*-value
Privacy and autonomy (12)	4.13	1,33		4.65	0.21	0.641
Windows and views (7)	5.00	0.00		4.03	0.35	0.034
Comfort and control (10)	4.35	0.35		3.77	0.67	0.352
Facilities (8)	5.00	0.00		4.37	0.32	0.077
Orientation and routing (14)	4.20	0.42		4.00	0.43	0.647
Interior (11)	4.80	0.14		4.00	0.27	0.032
Nature (7)	4.85	0.21		3.77	1.01	0.250
Staff (9)	4.55	0.21		3.83	0.12	0.015
Total	4.68	0.09		4.00	0.30	0.057

N = number of NHs; SD = standard deviation. OAZIS dementia categories range from [1] Strongly disagree to [5] Strongly agree

## Differences in health

The comparison of health between residents living in semi-open and closed NHs is shown in Table 3. After controlling for age, gender, and type of dementia, residents in semi-open NHs and residents in closed NHs had similar scores on all Qualidem subscales (*p*-values range from 0.280 to 0.730). The level of agitation based on the CMAI scores were also comparable between the two groups (*p* =.621). Cognitive functioning, as measured by the InterRai Cognition scores, was similar for residents in both semi-open and closed NHs (*p* =.327). Depression levels, assessed using the CSDD, were comparable between the two groups (*p* =.087). Mobility, as measured by the POMA, was similar for residents in both types of NHs (*p* =.870). Scores on ADL differed significantly between the two groups (*p* =.020) with a better ADL for residents living in closed NHs. This indicates greater independence in basic ADL compared to residents in semi-open NHs. However, after applying a correction for multiple testing, no significant differences were found in the level of ADL independence with a *p*-value threshold of less than 0.004.

**Table 3:** Comparison of health of residents living in closed and semi-open NHs

	Closed NHs			Semi-open NHs			*p*-value
	*n*	mean	SD	*n*	mean	SD	
Qualidem subscores							
Care relationship	63	15.86	5.01	61	14.79	4.82	0.315
Positive affect	63	14.11	4.62	61	13.03	4.36	0.218
Negative affect	63	6.46	2.64	61	6.90	2.48	0.485
Restless tense behaviour	63	5.48	3.21	61	4.84	3.07	0.435
Positive self-image	63	7.76	0.26	61	7.49	2.47	0.439
Social relations	63	10.78	4.97	61	10.30	4.73	0.730
Social isolation	63	6.76	2.60	61	6.92	2.28	0.597
Feeling at home	63	9.54	3.15	61	9.20	3.39	0.560
Having something to do	63	2.33	2.26	61	1.92	2.06	0.280
CMAI	63	48.48	19.67	61	47.66	13.92	0.621
InterRai Cognition	63	9.30	3.48	61	9.97	2.98	0.327
Barthel Index	63	7.94	5.67	61	10.28	5.18	0.020
CSDD	62	4.34	3.74	50	5.74	3.94	0.087
POMA	25	18.04	8.82	50	18.02	7.50	0.870

Grouping Variable: level of freedom (semi-open and closed). Group differences were tested with the ANCOVA, adjusted for age, gender and type of dementia

n: number of participants; SD: standard deviation. CMAI: Cohen-Mansfield Agitation Inventory; CSDD: Cornell Scale for Depression in Dementia; POMA: Performance-Oriented Mobility Assessment according to Tinetti

Corrected significance threshold: *p* <.004

## Discussion

This study compared residents with dementia living in semi-open NHs with residents living in closed NHs on several health dimensions. The two groups were similar in terms of age, gender, type of dementia, length of stay, length of diagnosis and type of care package. Further, the groups showed no significant differences regarding their health, including quality of life, agitation, cognition, and mobility. In contrast to the findings of this study, previous research indicated that more freedom of movement among a group of NH residents was related to improvements in their health [6, 13]. The closed and semi-open NHs in this study had similar scores on most environmental aspects. This may partly explain the absence of health differences between residents as well, although semi-open NHs did score better on ‘windows and views’, ‘interior’ and ‘staff’. Also, it is possible that people with dementia who move into a NH adjust to a new situation in which going outside is not a key part of their lived space. Theories of institutionalization describe how individuals may adapt their behaviors over time when entering an institutional setting [33]. This may depend on a person’s perceptions of a certain environment, as well as on the acceptance and internalization of accompanying roles as ‘a client’ or ‘a resident’. For instance, in a former study, residents with dementia who had lived in a closed NH before moving to an open NH, were surprised that the door was not locked and these residents had to be encouraged to go outside, because they still thought they were not allowed [9]. Also, in a recent study, NH residents seemed to take the closed units for granted and did not protest against the locked doors [34]. Another study found that depressive symptoms that occurred in newly admitted NH resident with dementia, decreased after several months because of their adaptation to the environment [35]. In their systematic review, Fitzpatrick and Tzouvara describe several strategies that newly admitted residents use to cope with their new living environment, including reframing and trying to ‘fit in’ by adopting the facility’s culture [36]. The current study’s finding of no health differences between NH residents in closed versus semi-open facilities, could be due to residents’ ability to adapt to their new environment regardless of their level of freedom. According to the theory of institutionalization, living in an institution can result in internalizing the norms and rules of the institution and disregarding one’s own initiative and personal values, especially in vulnerable populations [33]. Subsequently, institutionalization may involve behaviors such as apathy, lethargy and passivity, and a diminished sense of self and sense of purpose. Hence, even if residents would eventually adapt to living in a NH environment, it should not be an argument to keep doors shut and to limit their freedom of movement. Rather, it is important that residents are activated and encouraged to use their space to move around [12]. This may also apply to (semi-) open NHs, as transforming the NH culture towards deinstitutionalized and person-centered care takes time [37]. Hence, there is also a continuing need to challenge traditional perceptions and role expectations of living in a NH.

In their meta-synthesis, Førsund and colleagues described that although the outdoors was an important part of the lived space among people with dementia who lived at home, it was barely discussed when persons lived in a facility [11]. A lack of communication about residents’ freedom of movement could have multiple reasons. It may indicate that the organization does not recognize it as an important topic for discussion with residents. For instance, organizations may have fixed rules and regulations to keep residents with dementia inside for safety reasons [5]. Moreover, previous studies suggest that healthcare professionals experience a great sense of responsibility for keeping residents safe, and that providing more freedom of movement brings along anxiety and moral concerns [7, 38]. These concerns may also keep them from discussing the topic with residents. The Care and Coercion act (‘Wet Zorg en Dwang’ or ‘Wzd’ in Dutch), established in the Netherlands in January 2020, asks of organizations to evaluate and report situations in which clients resist care or do not consent with restrictive measures. Ideally this would include multidisciplinary consultation and conversations with clients and their representatives. However, previous studies suggest that changes in legislation do not immediately translate into changes in everyday care practice [39, 40]. Also, cultural norms and behaviors generally change slowly especially when informal and formal caregivers and organizations believe that they may put residents at risk by increasing their freedom, and are therefore keeping the doors closed [7].

Besides freedom of movement, multiple other factors have been described that may either facilitate or impede residents’ adaptation to their environment. For example, retaining autonomy, having one’s personal belongings in a new environment, and continuing or building new social relationships may support residents’ adaptation to the NH [41]. In particular for NH residents with dementia, enabling feelings of belonging and a ‘sense of home’ is key to their adaptation [11]. Although freedom of movement may be an important part of person-centered care, NHs are complex environments with many potentially influencing factors. The NHs in our study differed in multiple environmental aspects, also beyond the level of freedom of movement. Yet, the residents living in each type of nursing home did not differ significantly in terms of their health. More research is needed to untangle how different elements of the NH environment, including freedom of movement, affect NH residents’ overall health and wellbeing.

Research on daily activities in NHs shows that NH residents with dementia are largely inactive during the day [42, 43]. Their inactivity can, for instance, be due to physical or cognitive problems, caregivers’ attitudes toward giving residents freedom of movement or environmental aspects of the NH that make it impossible for residents to walk around [42]. Thereby, because of potentially confounding variables that are difficult to control for, it is complex to explain how the environment is affecting daily activities [44]. Nonetheless, NH residents with dementia can become increasingly dependent on others to stimulate them to go outside and help them to find their way around [11]. When there is insufficient help for these residents, they may be limited in their mobility and sense of freedom [45]. In the current study, we did not evaluate to what extent the physical environment and freedom of movement was used by residents. Even when NH environments provide freedom of movement, residents may not experience health benefits when sufficient support or guidance to use the physical space is lacking. In a cross-sectional study, it is not possible to provide a definitive explanation for these findings.

### Limitations

To our knowledge, this is the first cross-sectional study to explore differences in various health dimensions between residents with dementia living in semi-open and closed NHs. However, some limitations must be addressed. First, in addition to the level of freedom of movement, other physical and social environmental differences between the closed and semi-open NHs, e.g., the floor area and staff characteristics, could have influenced the residents’ health. Second, the results are based on the perspective of formal caregivers regarding the health of NH residents. Rigor of assessing health was established by having all the questionnaires (except for the POMA) completed by two formal caregivers each. Nonetheless, considering the perspectives of NH residents with dementia and their informal caregivers could have altered the results. Residents with dementia, for example, may rate their own quality of life as significantly higher than their formal caregivers would [46]. Third, this study did not consider how residents interact with their environment (i.e., how they used their living space), which could have influenced the relationship between freedom of movement and health of NH residents with dementia. Obtaining a comprehensive understanding of this complex relationship was limited by the cross-sectional design of this study. Controlled, longitudinal research would be necessary to establish a more conclusive understanding. Also, future studies should focus on whether and how residents are encouraged to utilize their freedom of movement, for instance, by conducting observations or interviews with formal caregivers. Lastly, in the current exploratory analysis, clustering of residents within NH units was not taken into account as the unit-level sample size was too small to perform a multilevel analysis. Future research should use larger samples at different levels to enable the use of more robust statistical models.

## Conclusion

This exploratory study found no significant differences in health between residents with dementia in semi-open and closed NHs. This contradicts previous findings suggesting that more freedom of movement may contribute to residents’ overall health. The current study, using a cross-sectional design, does not provide information on causality. Hence, further research, ideally utilizing longitudinal designs, is needed to demonstrate causal pathways and uncover underlying mechanisms.

## Data Availability

The dataset generated and/or analyzed during the current study is not publicly available due to the sensitive nature of the data but is available from the corresponding author upon reasonable request and after approval by the Ethics Review Board of Tilburg University.

## Abbreviations

NH: Nursing home
ADL: Activities of Daily Living
CMAI: Cohen-Mansfield Agitation Inventory
CSDD: The Cornell Scale for Depression in Dementia
InterRAI LFTC: InterRAI Long-Term Care Facilities System
POMA: Performance-Oriented Mobility Assessment according to Tinetti
ANCOVA: Analysis of covariance N: number
M: Mean
SD: Standard deviation
ZZP: Type of care package
